# Interleukin 8 Molecular Interplay in Allergic Rhinitis and Chronic Rhinosinusitis with Nasal Polyps: A Scoping Review

**DOI:** 10.3390/life15030469

**Published:** 2025-03-15

**Authors:** Romica Cergan, Ovidiu Berghi, Mihai Dumitru, Daniela Vrinceanu, Felicia Manole, Gabriela Cornelia Musat, Alina Lavinia Antoaneta Oancea, Crenguta Serboiu

**Affiliations:** 1 Anatomy Department, Carol Davila University of Medicine and Pharmacy, 050474 Bucharest, Romania; r.cergan@gmail.com; 2 Allergology Department, Saint Mary Clinics and Laboratories, 011013 Bucharest, Romania; oberghi@yahoo.com; 3 ENT Department, Carol Davila University of Medicine and Pharmacy, 050474 Bucharest, Romania; vrinceanudana@yahoo.com; 4 ENT Department, Faculty of Medicine, University of Oradea, 410073 Oradea, Romania; 5 ENT Department, Faculty of Dentistry, Carol Davila University of Medicine and Pharmacy, 020021 Bucharest, Romania; gabimusat@yahoo.com; 6 ENT Department, Elias University Clinical Emergency Hospital, Carol Davila University of Medicine and Pharmacy, 050474 Bucharest, Romania; dr.alina.oancea@gmail.com; 7 Histology and Molecular Biology Department, Carol Davila University of Medicine and Pharmacy, 020021 Bucharest, Romania; crengutas@yahoo.com

**Keywords:** interleukin, allergic rhinitis, sinusitis

## Abstract

The present scoping review underlines the molecular interplay between allergic rhinitis (AR), chronic rhinosinusitis with nasal polyps (CRSwNP), and interleukin-8 (IL-8). A query of PubMed database resulted in the inclusion of 34 articles in the final analysis of this scoping review. IL-8 is one interconnecting immune mediator in the physiopathology of AR and CRS. An influx of cytokines, such as interleukin (IL)-4 and IL-13, occurs from mast cells, four to six hours after the initial response signifying the development of the late-phase response allowing the entrance of eosinophils, basophils, and T-lymphocytes at the level of nasal mucosa. Chronic rhinosinusitis (CRS) is a chronic inflammatory disease that occurs in the mucosa of the nasal cavity and sinuses with two external phenotypes, but with molecular mechanisms that overlap with allergic rhinitis. Interleukin 8 induces neutrophil chemokinetic movement providing a chemotactic or directional cue. Clinical and fundamental studies established an implication of IL-8 in the disease mechanism of allergic rhinitis and CRSwNP. Moreover, there is still missing a randomized, large-cohort study with three patients groups (normal control, AR, CRSwNP) that analyzes the impact of IL-8 simultaneously. Future possible developments could focus on IL-8 as possible target for biologic treatments.

## 1. Introduction

Allergic rhinitis (AR) is an immune-mediated condition characterized by inflammation of the nasal mucosa, driven by the activation and interaction of various immune cells. In the United States and Europe, research estimates that 20–30% of adults are affected by AR. Clinical symptoms include nasal itching, sneezing, nasal obstruction or congestion, rhinorrhea (anterior or posterior), and sometimes a reduced sense of smell (hyposmia). Conjunctivitis occurs in over half of AR cases, presenting with eye itching, redness, and watering [1].

AR begins with an allergic immune response to inhaled allergens. In the nasal mucosa, dendritic cells act as antigen-presenting cells by binding allergens to specific major histocompatibility complex (MHC) class II molecules. This immune complex is recognized by Th0 lymphocytes, which, with the help of co-stimulatory molecules, differentiate into Th2 CD4+ lymphocytes. These cells produce cytokines like IL-4, IL-5, and IL-13, driving an IgE-mediated inflammatory response. This activation stimulates B-cell receptors, prompting B-cell differentiation into plasma cells that produce antibodies. Under the influence of IL-4, B lymphocytes switch from producing IgM to IgE. During pollen exposure, an antigen-specific IgE binds to high-affinity IgE receptors on mast cells and basophils, triggering the release of mediators such as histamine, leukotrienes, prostaglandins, and enzymes that activate matrix metalloproteinases, leading to tissue damage. Additionally, epithelial cell cytokines such as IL-25 and IL-33 enhance the Th2 immune response [2].

Recent studies highlight the involvement of other immune cells in AR, including ILC2, Tregs, B cells, B regulatory cells (Bregs), T follicular helper cells (Tfh), follicular regulatory T cells (Tfr), and LOX-1+ neutrophils [3]. A higher presence of CD44−CD62L+ Tregs has been associated with a Th1/Th2 imbalance and reduced immunosuppressive effects on Th2 cells [4]. The transcription factor FOXO1 regulates the differentiation of naïve T cells into IL-9-producing T cells, which may contribute to asthma-related symptoms and a pro-asthmatic phenotype in lung macrophages. Genetic variants affecting FOXO1 expression have been linked to AR development in studies conducted in Singapore and Malaysia [5].

In AR patients, mRNA expression levels of NLRP3 and IL-1β, along with IL-1β production in monocytes and macrophages, are significantly elevated compared to healthy controls [6]. Key remodeling factors such as vascular endothelial growth factor (VEGF), matrix metalloproteinases (e.g., MMP-9), tissue inhibitors of metalloproteinase-1 (TIMP-1), and TGF-β1 play crucial roles in tissue remodeling associated with AR. These factors are particularly pronounced in chronic rhinosinusitis (CRS) cases accompanied by AR compared to CRS without AR [7].

We aim to perform a scoping review of the current state of the art regarding the interplay between IL-8, AR, and chronic rhinosinusitis.

## 2. Material and Methods

The design of this scoping review followed these steps: formulating the research question, conducting a search for relevant studies, selecting studies through an iterative team-based approach, charting the data, and summarizing and reporting the results.

**Figure 1:**
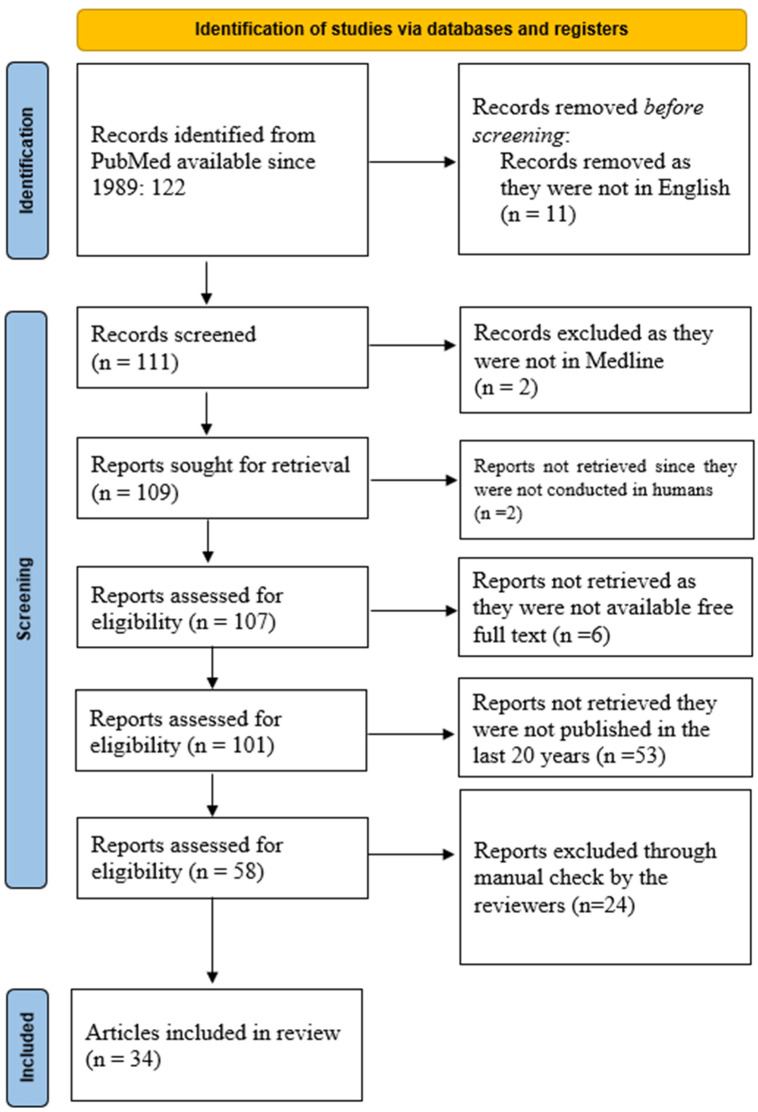
PRISMA diagram of the current scoping review about the importance of IL-8 in AR and CRSwNP.

The PubMed database was queried using the keywords “Interleukin 8” AND “Allergic Rhinitis” AND “Chronic Rhinosinusitis”, resulting in 122 manuscripts from 1989 onwards. Restricting the search to articles in English reduced the number to 111. A similar search in the Medline database yielded 109 articles, including 107 manuscripts on human subjects, of which 101 articles were available in full text. Of these, 58 articles were published in the last 20 years. The search results, summarized in Figure 1, were imported into an online cloud database for further analysis.

Screening for inclusion was carried out independently by two groups of two reviewers (O.B. and C.S.; M.D. and G.C.M.), who reviewed titles, abstracts, and full texts. Any discrepancies were resolved by a separate set of reviewers (R.C., A.L.A.O and D.V.). The entire process was remotely supervised by F.M., who was not affiliated with the same university center as the other reviewers, a measure taken to reduce potential bias.

After completing the manual screening, 34 manuscripts were included in the final analysis.

## 3. Results and Discussion

**Figure 2:**
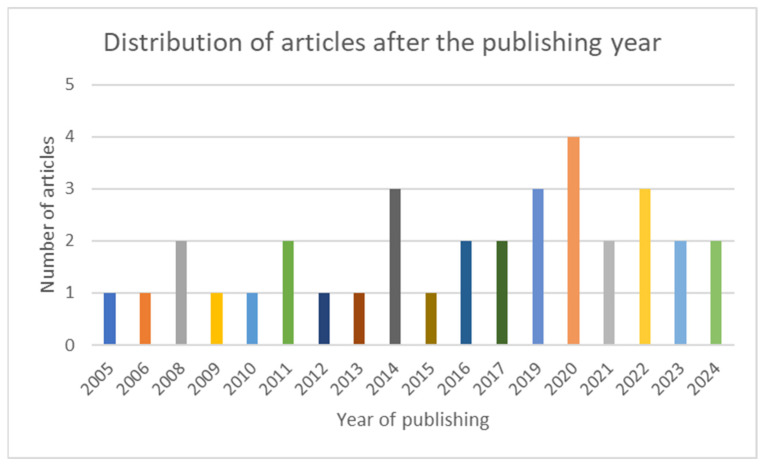
Chart showing the distribution of the included articles after the publishing year.

The articles included in the scoping review are grouped and summarized in three categories: latest data about IL-8, AR and IL-8, CRSwNP, and IL-8. In Figure 2, the chart presents the distribution of the included articles by year of publication.

### 3.1. Interleukin 8 (CXCL8)

IL-8, encoded by the IL8 gene, consists of four exons and three introns, located on chromosome 4 at locus q12-q21. It plays a key role in inducing neutrophil chemokinetic movement by providing chemotactic or directional cues [8]. Under normal physiological conditions, IL-8 is not expressed; however, its mRNA is massively induced in various tissue cells and leukocytes in response to inflammation [9].

Originally identified in 1987–1988 from stimulated cell culture supernatants, IL-8 was characterized as a low-molecular-weight protein with potent neutrophil chemoattractant properties. It acts as a monocyte- or lymphocyte-derived neutrophil chemoattractant and activating factor/peptide. IL-8 is produced and released by leukocytes and nearly all other cell types in response to endogenous or exogenous pro-inflammatory stimuli.

CXCL8, another name for IL-8, interacts with chemokine receptors CXCR1 (IL-8RA/IL-8R1), CXCR2 (IL-8RB/IL-8R2), and the atypical chemokine receptor ACKR1. It regulates endothelial adhesion, chemotaxis, and the activation of various leukocytes, including IL-13/IL-4-stimulated monocytes, subsets of CD8+ T-lymphocytes, mast cells, and non-immune cell migration. Additionally, it stimulates corneal neovascularization and promotes angiogenesis, similar to other ELR+ chemokines.

Elevated levels of IL-8 are commonly observed in inflamed tissues and the blood circulation of individuals with several inflammatory diseases, including asthma, idiopathic pulmonary fibrosis, transplant-related complications, ischemia–reperfusion injury, arthritis, multiple sclerosis, kidney diseases, psoriasis, systemic lupus erythematosus, inflammatory bowel diseases, and sepsis [10].

### 3.2. Allergic Rhinitis and IL-8

About two decades ago, the first articles described the role of interleukin 8 in the mechanism of allergic rhinitis. Kramer and colleagues collected nasal secretions of 13 individuals with allergic rhinitis gained over a period of 8 h after nasal allergen challenge. Interleukin 8 peaked in the early-phase reaction, reaching its maximum 10 min after allergen challenge, with values dropping below before provocation after 30 min and steadily increasing in the late-phase reaction [11].

Ural A. and colleagues found that IL-8 levels in polypoid tissue were elevated in 35 patients with intranasal polyposis (20 allergic and 15 non-allergic) compared to 12 healthy controls. Notably, IL-8 levels were similar between allergic and non-allergic patients, suggesting local IL-8 synthesis in the nasal mucosa could occur through a non-IgE-mediated mechanism [12].

Wu, Bing, and Jingping analyzed gene expression profiles in nasal polyp (NP) tissues from six patients with AR-associated NP. Using oligonucleotide chip techniques, they found significant upregulation of IL-8 in NP tissues and the mucosa of the inferior nasal concha in AR patients compared to normal nasal mucosa [13].

Shazly and Lefebvre studied 16 AR patients sensitized to aeroallergens confirmed by skin tests or radioallergosorbent tests. A nasal allergen challenge showed increased IL-8 secretion by NK cells following IL-15 treatment, revealing a novel inflammatory interaction between NK cells and eosinophils through the IL-15/IL-8 axis [14].

A research team led by Simona Lavinskiene evaluated neutrophil chemotaxis after IL-8 stimulation in 47 nonsmoking adults, including 18 patients with mild-to-moderate/severe persistent allergic rhinitis, 14 with intermittent or mild-to-moderate persistent allergic asthma, and 15 healthy controls. Neutrophil response to IL-8 was significantly increased in patients with allergic rhinitis and asthma, with a stronger effect observed in asthma patients. Serum IL-8 levels were higher in asthma patients than in those with rhinitis or healthy controls, both at baseline and 7 and 24 h after allergen challenge [15].

In a study led by Mey Fan-Lee, 64 participants were divided into three groups based on allergic severity: those with persistent asthma and rhinitis (AS), those with allergic rhinitis only (AR), and nonallergic controls (NA). Serum IL-8 levels were significantly higher in the AS group compared to the AR and NA groups, indicating a correlation between IL-8 levels and clinical severity [16].

Immacula Ventura and her team studied neutrophils from 15 pollen-allergic patients undergoing immunotherapy (IT), 10 untreated pollen-allergic patients, and 10 healthy controls. Neutrophils stimulated in vitro with LPS showed IL-8 production through an NF-κB-dependent pathway. Immunotherapy reduced IL-8 release by LPS-stimulated neutrophils in allergic asthmatic patients [17].

Lavinskiene et al. also explored the relationship between sputum neutrophilia and peripheral blood neutrophil chemotactic activity following bronchial allergen challenges in asthma patients. Their study included 36 nonsmoking adults—15 with intermittent or mild-to-moderate persistent allergic asthma, 13 with mild-to-moderate/severe persistent allergic rhinitis, and 8 healthy controls. Bronchial challenge with varying concentrations of a D. pteronyssinus allergen resulted in significantly elevated sputum IL-8 levels at 7 and 24 h in allergic asthma and rhinitis patients compared to healthy subjects. IL-8 levels were particularly higher in asthma patients than in those with rhinitis [18].

Masanari Watanabe conducted a study on the impact of Asian dust storms (ADS) on asthma. The study involved 184 adult outpatients with asthma residing in four locations in western Japan. Of these, 112 patients were deemed eligible for analysis. ADS, originating from the deserts of Mongolia, northern China, and Kazakhstan, typically disperse dust across East Asia from spring to late autumn. The study found that ADS were linked to an increased risk of asthma exacerbations. Airborne particles collected during ADS events in western Japan (but not soil from the ADS origin) were shown to increase IL-8 secretion in THP-G8 cells. THP-G8 cells, derived from the THP-1 cell line, are engineered to express luciferase genes regulated by the IL-8 and GAPDH promoters. IL-8 concentrations in supernatants of THP-G8 cells stimulated with various agents were as follows: vehicle (0.03 ± 0.002 mg/mL), LPS (38.1 ± 1.7 mg/mL), CJ-1 soil (0.30 ± 0.004 mg/mL), and ADS particles (29.7 ± 1.5 mg/mL). These findings suggest that ADS exposure may exacerbate asthma by enhancing IL-8 transcriptional activity [19].

In 2015, Lauren A. Lawrence published research on cytokine secretion in response to fungal exposure. The study demonstrated that exposure to Aspergillus fumigatus and Alternaria alternata increases the secretion of pro-inflammatory cytokines IL-6 and IL-8. Human sinonasal epithelial cells (HSNECs), which form the first barrier against environmental allergens and toxins, play a crucial role in airway inflammation. HSNECs were cultured from sinus tissue explants obtained during surgery from control patients and those with chronic rhinosinusitis with nasal polyps (CRSwNP). Cells exposed to A. fumigatus and A. alternata showed increased IL-6 and IL-8 secretion, particularly in CRSwNP patients [20].

Additionally, Shino Shimizu investigated the role of HMGB1 (High-Mobility Group Box 1) in upper-airway inflammation. HMGB1, known for its association with acute inflammatory conditions like sepsis and chronic diseases such as airway disorders, was studied in 32 patients with chronic rhinosinusitis with nasal polyps (CRSwNP), allergic rhinitis, and control subjects. Nasal secretions were analyzed to assess HMGB1’s presence and its role in inflammation. HMGB1 was found to stimulate IL-6 and IL-8 production in cultured nasal epithelial cells via TLR4 signaling. Blocking TLR4 with specific antibodies significantly reduced IL-6 and IL-8 secretion induced by HMGB1 [21].

An international collaboration led by S. Gilles-Stein, investigated patients sensitized to birch and grass pollen or sensitized only to grass pollen (but not birch) from Germany. These patients underwent skin prick testing with the allergen alone, allergen plus Bet-APE (aqueous pollen extracts of birch, B. Pendula < 3 DA), or allergen plus pre-identified candidate substances from the low molecular weight pollen fraction. Larger weals were produced when allergens were tested in combination with the low molecular weight compounds from pollen or with candidate pollen-associated lipid mediators. Nasal allergen challenges were performed with the allergen alone or allergen plus Bet-APE < 3 DA. Compared to patients challenged with allergen only, there was a significant increase in IL-8 and IgE in the nasal-lining fluids of allergic patients challenged with allergen plus Bet-APE < 3 DA. Bet-APE < 3 kDA also led to increased local IL-8 release, nasal secretion production, and reported symptoms during nasal allergen challenges [22].

A.B. Ozturk investigated the role of diesel exhaust particles (DEPs) in atopy and the pathogenesis of allergic upper-airway diseases. The hypothesis was that the DEPs might activate oxidative stress pathways, such as nuclear factor (NF-κB), activator protein-1 (AP-1), and the mitogen-activated protein kinase (MAPK) pathway, in macrophages and human bronchial epithelial cells (BECs). Eighteen volunteers participated in the study, consisting of six controls, five with allergic rhinitis, and seven with primary nasal polyposis. Nasal epithelial cells (NECs) were cultured and incubated with 0–100 µg/mL DEP for 24 h. NECs from subjects with atopic polyps released significantly higher amounts of IL-8 and RANTES. While 50 µg/mL DEP induced RANTES release in NECs from patients with allergic rhinitis, 100 µg/mL DEP decreased IL-8 levels in NECs from both control and allergic rhinitis subjects [23].

Emel Ceylan and colleagues evaluated the levels of interleukin 8 (IL-8) and interleukin 13 (IL-13) in a study of 120 clinically stable asthma patients (71 females and 49 males) and 35 healthy controls (19 females and 16 males) who were similar in age, gender, and body mass index. Allergic rhinitis coexisted as a comorbidity in 15% of the patients. IL-8 levels were found to be significantly higher in asthma patients with comorbidities compared to those without comorbidities [24].

A team led by Ricardo Gaspar studied the role of pollen proteases in allergic disorders. Pollen proteases, which contain multiple allergens and proteolytic enzymes, are important sensitizing agents in Southern Europe (e.g., Flea European, Dactylis glomerata, and Protea judaica). These proteases degrade intercellular adhesion proteins, facilitating the detachment of human epithelial cells and allergen delivery across the epithelium. Pollen proteases from Chenopodium album, Plantago lanceolata, and Eucalyptus globulus induced pro-inflammatory cytokine production of IL-6 and IL-8 in Calu-3 human airway epithelial cells, which form functional tight junctions in vitro [25].

Mehmet Gokkaya coordinated an international investigation in which the kinetics of humoral immune response were compared in subjects with seasonal allergic rhinitis (SAR) and subjects without allergy. The subjects were tested for cross-sectional and interseason differences in levels of serum and nasal, total, and Betula verruca 1–specific immunoglobulin isotopes, immunoglobulin-free light chains, cytokines, and chemokines. At the start, a total of 50 candidates underwent an initial screening procedure to confirm eligibility (questions on self-reported symptoms, skin prick test, and a serum IgE test). Analyses were performed on seven patients with SAR and seven subjects without allergies. Differences in the IL-8 level were apparent only in seasons with IL-8 levels higher in subjects without allergies. Nasal levels of IL-8, IL-33, Betula verruca 1–specific IgG4 (sIgG4), and Betula verruca 1–specific IGE (side) antibodies were found to be predictive for seasonal symptom severity (predictive biomarkers for pollen-specific symptom expression, irrespective of atopy) [26].

Xiangting Bu and colleagues, conducted a study on serine protease inhibitors (Serpins), a family of homologous proteins. Increased expression of SerpinB3 and B4 has been observed in inflammatory conditions such as asthma, atopic dermatitis, and psoriasis. The study enrolled 97 participants, including 36 patients with ECRSwNP, 30 patients with non-ECRSwNP, and 31 healthy controls. The results showed a significant downregulation of CXCL8 when stimulated with SerpinB3 and B4 compared to the untreated group. Additionally, the knockdown of SerpinB3 and B4 led to an upregulation of CXCL8 expression and an increase in IL-8 secretion in the supernatant [27].

**Figure 3:**
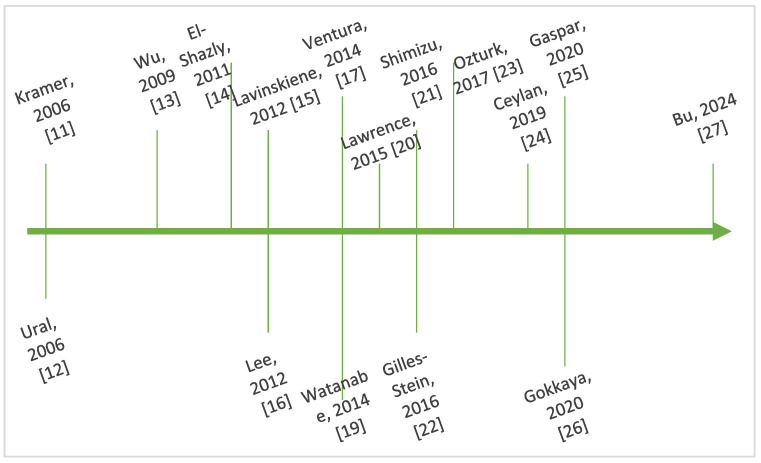
Timeline showing key steps in the chronology of research regarding IL-8 and AR [11,12,13,14,15,16,17,19,20,21,22,23,24,25,26,27].

Figure 3 outlines key steps in the chronological evolution of the research regarding the interplay between IL-8 and AR.

### 3.3. Chronic Rhinosinusitis with Nasal Polyps and IL-8

According to the 2020 European Position Paper on Rhinosinusitis and Nasal Polyps (EPOS), CRS, with or without nasal polyps, is characterized by the presence of at least two of the following symptoms: (a) nasal congestion or nasal discharge (including anterior or posterior nasal drip), (b) facial pain or pressure, and (c) reduced or lost sense of smell. Nasal polyposis typically presents as bilateral, benign growths and accounts for about 25–33% of all CRS cases [28].

K. Kostamo et al. noted that interleukin 8 was observed with divergent values in patients with CRSwNP. The MMP-8, IL-8, and TNF-α levels were measured from 13 patients undergoing a paranasal sinus operation and from 19 healthy volunteers from preoperative nasal lavage at University Central Hospital Helsinki. In the CRSwNP patients, the concentration of IL-8 was significantly increased in patients when compared to controls. The IL-8 and MMP-8 made a cytokine–proteinase cascade in CRSwNP pathogenesis [29].

Yih Jeng-Tsai evaluated bradykinin (BK), a potent inflammatory mediator and stimulus of the expressions of interleukin-1, IL-6, IL-8, and eotaxin in fibroblasts, in lower- but not in upper-airway diseases in human lung/bronchial fibroblasts. A total of 12 patients with CRSsNP were recruited, and 10 patients who came into correct nasal septal deviations were recruited as a control group (Shin Kong Wu Ho-Su Memorial Hospital, Taipei, Taiwan). BK upregulated CXCL8 chemokine mRNA and protein expression, proliferation, and pro-inflammatory molecule expression in CRSsNP-derived fibroblasts through B2R activation [30].

Prostaglandins, leukotrienes, and thromboxanes—metabolites of arachidonic acid—play a key role in mucosal inflammatory responses, such as those observed in allergic rhinitis and chronic rhinosinusitis. These molecules activate various cell types, including T lymphocytes, epithelial cells, endothelial cells, and others. A study conducted at Shin Kong Wu Ho-Su Memorial Hospital in Taipei, Taiwan, included 14 patients with chronic rhinosinusitis with nasal polyps (CRSsNP) and 13 controls with nasal septal deviations. Compared to the control group, CRSsNP specimens exhibited stronger expression of CXCL1 and CXCL8 (highlighted by deep-red staining) in the submucosal stroma, vessel walls, and glands. Stimulation by TXA2 activated PI-3K, PKA, and PKCμ/PKD, which led to CREB phosphorylation and cross-talk with the PI-3K pathway, promoting CXCL1/8 mRNA and protein expression [31].

In a study by Seung Heon-Shin, the recognition of fungal pathogens by pathogen recognition receptors (PRRs) was found to activate antifungal responses, such as the production of pro-inflammatory cytokines, degranulation of inflammatory cells, and differentiation of Th1 cells in nasal polyps and inferior turbinate fibroblasts. Primary nasal fibroblasts were isolated from seven patients with chronic rhinosinusitis with nasal polyps. *Alternaria* stimulation (at concentrations of 100 and 50 mg/mL) significantly increased IL-8 production after 8, 24, and 48 h of exposure. This suggests that *Alternaria* induces IL-8 production from nasal polyp fibroblasts, triggering a cycle of neutrophil infiltration and IL-8 generation by epithelial cells, fibroblasts, and glandular cells in inflamed areas of the nasal cavity [32].

Jung Sun-Cho investigated the role of prostaglandin E2 (PGE2) in increasing IL-6 and IL-8 levels across different cell types. Fibroblasts derived from eight patients who underwent endoscopic sinus surgery for chronic rhinosinusitis with nasal polyps (CRS with NP) were cultured with PGE2. The study found that PGE2 significantly upregulated both mRNA and protein levels of IL-6 and IL-8 in nasal polyp-derived fibroblasts (NPDFs) [33].

Huriaty et al. conducted a cross-sectional comparative study carried out on 15 samples of mucosal brushing and polyp tissue regarding expressions of IL-5, IL-8, IL-17A, and TGF-β on mucosa, which were measured using the most convenient and non-invasive examination tool in recurrent CRSwNP. Only IL-8 had a significant relationship between mucosa and tissue with moderate positive correlation [34].

Jingyun Li enrolled 333 adult participants for a study aimed to explore the relationship between DNA methylation in the IL8 proximal promoter and chronic rhinosinusitis (CRS). The analysis revealed significant reductions in DNA methylation at CpG sites 1, 2, and 3 in the IL8 proximal promoter in nasal epithelial cells of patients with CRSwNP compared to those with CRSsNP and control subjects. Additionally, IL-1β and TNF-α were found to significantly increase IL8 expression, which was associated with a reduction in methylation at the CpG3 site [35].

Rosati et al. conducted a study on the inflammatory profile of 44 patients with long-term recurrent and non-recurrent CRSwNP. They concluded that IL-8 expression did not correlate with the long-term recurrence rate of CRSwNP [36].

A study by do Amaral, involving 27 patients with CRSwNP without aspirin intolerance, 12 with aspirin intolerance, and 35 controls with no nasal inflammatory conditions, found lower IL-8 concentrations in CRSwNP tissue. However, no significant correlation was found between IL-8 levels and eosinophilic polypoid conditions [37].

**Figure 4:**
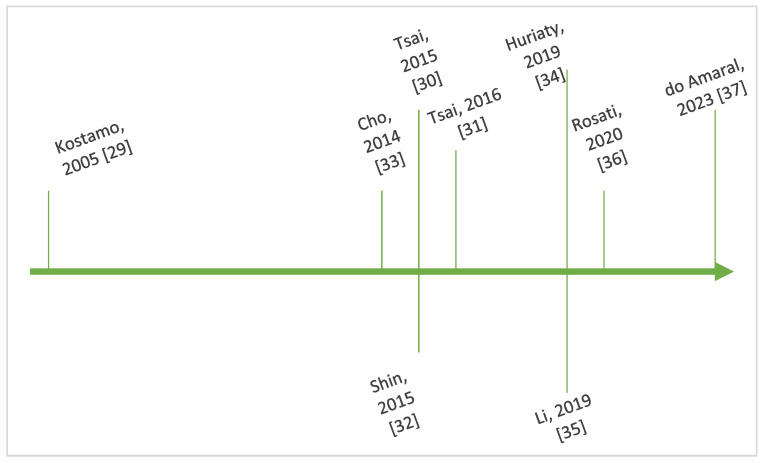
Timeline showing key steps in the chronology of research regarding IL-8 and CRSwNP [29,30,31,32,33,34,35,36,37].

Figure 4 outlines key steps in the chronological evolution of the research regarding the interplay between IL-8 and CRSwNP.

## 4. Limitations and Future Perspectives

There are some limitations to the present scoping review such as articles that were not available free full text for analysis. The heterogeneity of the research posed a challenge, as patients came from diverse continents—North America, Asia, and Europe—while South America, Africa, and Australia had no studies. North America was represented only by a few clinics in the United States, while Asia included studies from China, Japan, and Korea. In Europe, research came from Lithuania, Germany, Spain, Portugal, Finland, and Turkey. This led to issues of representation both across continents and within specific regions, potentially contributing to ethnic misrepresentation.

Another area requiring further exploration is the geographical variability in immune responses among different ethnic groups. This is particularly complex in today’s era of increased global migration and subsequent exposure to diverse immune triggers [38].

The number of patients included in studies varied significantly, ranging from 12 in Wu’s study to 112 in Watanabe’s. Furthermore, different methodologies were used to assess IL-8, including immunologic, genetic, transcriptomic, and enzymatic assays. Samples were obtained from nasal challenges, sinus and nasal tissue explants, serum, and nasal secretions, making systematic comparisons difficult. Local allergic rhinitis is another topic that requires further investigation regarding the molecular interplay and the connection with interleukin 8. This new approach is focusing on personalized medicine in allergies [39]. One limitation of our analysis is that we focused on IL-8 in relation to allergic rhinitis (AR) and chronic rhinosinusitis (CRS), excluding asthma within the broader context of united airway disease theory. Since Crossman introduced the idea of “the same airway, the same disease” in 1997, research in this field has grown, and the connection between the upper- and lower-airways is now widely recognized. While it is challenging to delineate the specific mechanisms of interaction between asthma and various upper-airway conditions like AR and CRS through a single theory, several overlapping mechanisms may contribute to this pathological phenomenon. United airway disease (UAD) describes the coexistence of upper- and lower-airway diseases as a unified entity, emphasizing their shared pathological traits. Increasing evidence highlights the complexity of UAD, which includes multiple phenotypes and endotypes as well as various respiratory diseases. The coexistence of asthma and chronic rhinosinusitis with nasal polyps (CRSwNP) is frequently noted; approximately 40% to 70% of patients with CRSwNP also experience asthma, which correlates with more severe sinonasal symptoms. Conversely, CRSwNP is present in 10% to 30% of mild asthma cases but can rise to 70% to 90% among those with severe asthma, often associated with poorer outcomes. Common pathophysiological mechanisms underpinning CRSwNP and asthma exist, with the type 2 (T2) endotype being the most prevalent. Acknowledging that a single biomarker may not capture the complexity of UAD, recent studies have explored combinations of biomarkers to create composite scores that could enhance predictive accuracy. Endotypes for airway diseases include type 1 (mediated by IFN-γ, IL-2, and lymphotoxin-α), type 2 (involving IL-4, IL-13, IL-5, and immune cells like eosinophils and mast cells), and type 3 (driven by neutrophilia and TH17 cells). This focus aligns with our expertise in allergology and ENT, aimed at examining the role of IL-8 in upper-respiratory inflammatory responses [40,41]. Applying Artificial Intelligence (AI) to the field of allergies helps investigators expand their understanding of disease pathogenesis, improve diagnostic accuracy, enable prediction for treatments and outcomes, and for drug discovery. Incorporating data regarding interleukin 8 in AI algorithms can accelerate the research process [42]. Biological treatment is the future for the management of this pathology. Current biological treatment compounds are focusing on interleukins (IL) IL-4, IL-5, and IL-13. Interleukin 8 could be a future candidate for the selective action of future biologic treatment [43]. There are other allied specialties that could benefit from extended research of the IL-8 molecular interplay. Current data analyzed the implications of IL-8 related mechanisms in the dry eye disease (DED) and ocular allergy (OA). IL-8 is another key chemokine that has been consistently increased in DED patients. Moreover, IL-8, IL-2, IFN-γ, and EGF may represent biomarkers of disease gravity in DED [44]. The possible clinical application of specific cytokines and chemokines contributing to tumorigenesis and the clinical outcome of several cancers has been reported. Pro-inflammatory cytokines IL-6 and IL-8 are potential biomarkers for cancer pathogenesis and could serve as markers of disease progression [45]. Sports medicine is another domain where further analysis of IL-8 could offer insight regarding the prevalence of exercise-induced bronchoconstriction (EIB) and also to investigate the effect of myokines in the performance of marathon runners [46]. The recent COVID-19 pandemics accelerated the research of the possible notable connection between IL-8 and viral responses, with circulating levels of IL-8 and soluble IL-2Rα being strongly associated with the duration of illness in patients with severe COVID-19 [47]. To better understand IL-8’s role in the atherosclerotic process and explore evidence supporting its causal involvement, cardiologists have examined the relationship between IL-8 levels and carotid intima-media thickness (c-IMT), a marker of vascular remodeling indicative of subclinical atherosclerosis. Elevated plasma IL-8 levels were found to correlate with increased c-IMT, suggesting a link to subclinical atherosclerosis [48]. Macrolides, commonly used as anti-inflammatory agents in respiratory conditions, have shown an off-target effect of inhibiting IL-8. This effect is thought to contribute to the reduced airway inflammation observed in patients undergoing chronic azithromycin therapy for airway diseases, such as chronic rhinosinusitis, where IL-8 levels correlate with disease severity and neutrophil infiltrates [49]. IL-8 also plays a role in lupus nephritis, a rare autoimmune disorder. In this condition, inflammatory cytokines like IL-6, IL-8, IL-1β, IFNα, IFNγ, and IP-10 are elevated in serum, and the transcriptional levels of interferon-stimulated genes are significantly higher [50]. Additionally, in Guillain–Barré syndrome (GBS) and chronic inflammatory demyelinating polyneuropathy (CIDP), IL-8 levels in cerebrospinal fluid (CSF) can help distinguish between the two. Patients with CSF IL-8 levels exceeding 70 pg/mL are more likely to have acute inflammatory demyelinating polyneuropathy (AIDP) rather than acute-onset CIDP, suggesting its potential as a diagnostic marker [51].

The implication of interleukin 8 in CRSsNP was also investigated. A search on PUBMED identified two relevant articles—one in Russian and one in Chinese—but they were excluded due to the lack of English content.

Future genetic studies are expected to provide more detailed insights into the risk of developing chronic rhinosinusitis (CRS). This section presents several relevant articles, though it does not explore the topic as in-depth as other parts of this article.

Jiang and colleagues examined the association between single nucleotide polymorphisms (SNPs) and CRS. The objective of their study was to assess the relationship between polygenic risk scores (PRSs) for nasal polyps (NP) and the risk of CRS, with or without NP. The study analyzed data from 535 individuals with CRS and 5350 control subjects from the Taiwan Precision Medicine Initiative project. Four PRSs for NP—PGS000933, PGS000934, PGS001848, and PGS002060—sourced from the UK Biobank were evaluated in these participants. Among them, PGS002060 demonstrated the highest area under the curve (AUC), measuring 0.534 for CRSsNP prediction and 0.588 for CRSwNP prediction. Compared to individuals in the lowest PRS category, those in the highest quintile of PGS002060 had a 1.48-fold increase in odds of CRSsNP (*p* = 0.003) and a 2.32-fold increase in odds of CRSwNP (*p* = 0.002). Notably, in female CRSwNP patients, the odds of developing CRSwNP increased 3.01 times in the highest quintile compared to the lowest PRS category (*p* = 0.009) [52].

Sivrice conducted a study to examine the relationship between angiotensin-converting enzyme (ACE) insertion/deletion gene polymorphism and CRS. The study included 52 cases with nasal polyps and 139 control subjects. Statistically significant differences in genotype and allele distribution were observed between the CRSwNP group and the control group (*p* = 0.015 and *p* = 0.003, respectively). However, no significant differences in genotype distribution were found in the CRSwNP group regarding NSAID allergy, asthma, or NSAID-exacerbated respiratory disease (*p* = 0.645, *p* = 0.660, and *p* = 0.095, respectively) [53].

Camargo conducted a pilot study evaluating the association between the VEGF-A (rs28357093) genetic variant and GSTM1/GSTT1 deletion polymorphisms in CRSwNP susceptibility. A case–control study was performed with 61 individuals diagnosed with CRSwNP and 100 healthy controls. VEGF-A (rs28357093) and GSTM1/GSTT1 deletion polymorphisms were genotyped using RFLP-PCR and SYBR Green real-time PCR, respectively. Individuals with allergic rhinitis carrying the AC genotype (rs28357093) had a 4-fold-increased risk of developing CRSwNP (OR = 4.20, 95% CI = 1.31–13.50; *p* = 0.015) [54].

## 5. Conclusions

Respiratory allergies are mostly Th2-mediated. The implication of Th1 and Th17 cells has been demonstrated recently. Interleukin 8, a chemokine known for its role in neutrophil chemotaxis, was found in high levels in patients with allergic rhinitis and CRSwNP in some articles and without correlation in others. Future studies are necessary to clear the role of this mediator in respiratory allergies. IL-8 could become another target for biological treatment also. Moreover, there is a lack of large-cohort, randomized, controlled studies analyzing the importance of IL-8 in the debut, development, and evolution of the pathology chain involved in AR and CRS. We hope to grow the awareness of fellow allergology specialists, ENT specialists, pneumologists and others, to this field of research development.

## Data Availability

All data are available upon request from the corresponding author.

## Abbreviations

The following abbreviations are used in this manuscript:

IL-8	Interleukin 8
AR	Allergic Rhinitis
CRS	Chronic Rhinosinusitis
MHC	Major Histocompatibility Complex
Bregs	B regulatory cells
Tfh	T follicular helper cells
Tfr	Follicular regulatory T cells
VEGF	Vascular endothelial growth factor
MMP-9	Matrix metalloproteinase 9
TIMP-1	Tissue inhibitors of metalloproteinase-1
NP	Nasal polyps
NA	Nonallergic controls
IT	Immunotherapy
ADS	Asian dust storms
HSNECS	Human sinonasal epithelial cells
HMGB1	High-Mobility Group Box 1
DEP	Diesel exhaust particles
AP-1	Activator protein-1
MAPK	Mitogen-activated protein kinase
BECs	Bronchial epithelial cells
NECs	Nasal epithelial cells
IL-13	Interleukin 13
SAR	Seasonal allergic rhinitis
Serpins	Serine protease inhibitors
EPOS	European Position Paper on Rhinosinusitis and Nasal Polyps
BK	Bradykinin
PRRs	Pathogen recognition receptors
PGE2	Prostaglandin E2
NPDFs	Nasal Polyp-derived fibroblasts
AI	Artificial Intelligence
DED	Dry eye disease
OA	Ocular Allergy
EIB	Exercise-induced bronchoconstriction
c-IMT	Carotid intima-media thickness
GBS	Guillain-Barre syndrome
CIDP	Chronic inflammatory demyelinating polyneuropathy
CSF	Cerebrospinal fluid
AIDP	Acute inflammatory demyelinating polyneuropathy
